# Departures from the Energy-Biodiversity Relationship in South African Passerines: Are the Legacies of Past Climates Mediated by Behavioral Constraints on Dispersal?

**DOI:** 10.1371/journal.pone.0133992

**Published:** 2015-07-24

**Authors:** Guillaume Péron, Res Altwegg

**Affiliations:** 1 Statistics in Ecology, Environment and Conservation, Department of Statistical Sciences, University of Cape Town, Rondebosch 7701, Cape Town, South Africa; 2 African Climate and Development Initiative, University of Cape Town, Rondebosch 7701, South Africa; University of Colorado, UNITED STATES

## Abstract

Legacies of paleoclimates in contemporary biodiversity patterns have mostly been investigated with global datasets, or with weakly dispersive organisms, and as a consequence been interpreted in terms of geographical or physical constraints. If paleoclimatic legacies also occurred at the regional scale in the distributions of vagile organisms within biomes, they would rather suggest behavioral constraints on dispersal, i.e., philopatric syndromes. We examined 1) the residuals of the regression between contemporary energy and passerine species richness in South African biomes and 2) phylogenetic dispersion of passerine assemblages, using occupancy models and quarter-degree resolution citizen science data. We found a northeast to southwest gradient within mesic biomes congruent with the location of Quaternary mesic refugia, overall suggesting that as distance from refugia increased, more clades were lacking from local assemblages. A similar but weaker pattern was detected in the arid Karoo Biomes. In mobile organisms such as birds, behavioral constraints on dispersal appear strong enough to influence species distributions thousands of years after historical range contractions.

## Introduction

Macroecological patterns theoretically result from the interaction of biogeographic history and evolutionary events [1] on the one hand, and contemporary ecological constraints [2] on the other hand. The extent to which evidence for the former is masked by the latter is currently subject to debate [3–5]. In particular, locally-available energy constrains both contemporary community carrying capacity [6,7] and long-term rates of speciation and extinction [8,9], so both pathways could shape observed species richness patterns [10]. For this reason, the residuals of the regression between species richness and energy should harbour phylogeographic signals of interest. Regions whose climate remained relatively stable during the Quaternary (“Quaternary refugia” [11,12]) are expected to hold more species than climatically unstable regions [13,14]. We hereafter refer to this shortfall below the potential number of species as “species debt”, as opposed to “species build-up” in refugia. Another metric of interest to study “Quaternary legacies” is phylogenetic dispersion, which quantifies how the average phylogenetic distance between the taxa composing local assemblages compares to the expectation based on null models [15]. Refugia have theoretically had a longer time to reach or approach community equilibrium via competitive exclusion, than the rest of the landscape. Assuming competition is stronger between related species [16,17], assemblages in refugia should therefore exhibit more phylogenetic over-dispersion than non-refugia assemblages of similar species richness (see however [18]). In addition, assuming that species’ dispersal abilities or dispersal behaviours are phylogenetically conserved [19,20], similar species are expected to re-colonize lost ground in a similar fashion after climate-driven range contractions. This hypothesis that dispersal tendencies are phylogenetically conserved is rather well supported by data from Amazonian forest fragments [21] and Andean endemism patterns [22], and in the African-Palearctic flyway, by the occurrence of resident clades that are lacking from or are sparsely represented at northernmost latitudes (Malaconotidea, Cisticolidae, Cettidae). We coin the phrase “philopatric syndromes” for the above-mentioned behavioural constraints on dispersal. Philopatric syndromes are quite common in birds [23]. For example black-headed gulls *Chroicocephalus ridibundus* exhibit reluctance to move between colonies separated by distances two orders of magnitude smaller than the distance they travel each year during seasonal migration [24]. In a macroecological context, philopatric syndromes may derive from the evolutionary loss of dispersal tendencies during periods of range contractions [25], leading to species build-up in refugia. Alternatively, they can be linked to micro-habitat specialization: refugia are more diverse floristically and, often, topographically [13,26] and they thus offer micro-habitats which may be lacking in the rest of the biome, preventing the spread of species that diverged in refugia. In brief, based on the above frame of working hypotheses, we predicted that refugia should be more species-rich and phylogenetically over-dispersed than the rest of their respective biomes, and that both species richness and phylogenetic dispersion should decrease as distance from refugia increases.

In Africa, most previous studies of Quaternary legacies had a continental scope [5,14,27], or focused on weakly dispersive forest-understorey endemics of the Eastern Arc Mountains [28,29]. In both cases, isolation by geography (distance, relief, rivers) could be invoked to explain Quaternary legacies. Here we set out to investigate a situation in which distances are moderate compared to the dispersal abilities of the study organisms, and thus Quaternary legacies if observed would be explained by philopatric syndromes. We focused on the passerine birds of South Africa, Lesotho and Swaziland (South Africa hereafter; 305 indigenous passerine species following the Clements checklist version 6.8 [30]). Our objective in this study was to 1) review the literature to produce predictions about Quaternary refugia in each of the nine biomes in South Africa [31], and 2) look for corresponding patterns in passerine distribution, thereby producing independent information supporting or contradicting current understanding of refugia and their locations in Southern Africa, as well as supporting our set of working hypotheses about the formation of species build-up in South African passerine assemblages. Importantly, the need for quantitative ways to measure how reliable species distribution data are and account for variation in sampling effort has become a growing concern in macroecology [32,33]. We corrected our stacked species distribution models (SDM; [34]) for imperfect sampling using occupancy models [35], and used them to compute biodiversity metrics at the quarter degree resolution across South Africa for passerines (305 species), using data from the Southern African Bird Atlas Project (SABAP; [36]), a citizen science project (crowdsourced data).

## Material and Methods

### Citizen science and occupancy models

By harnessing the sampling effort of thousands of participants, citizen-science projects can sample the distributions of whole biological classes at large spatial scales and fine spatial resolution, making it possible to tackle complex issues such as the separation of Quaternary legacies from the effect of contemporary conditions. However, citizen-science databases come with inherent problems [37,38]. First, sampling effort is necessarily biased towards areas where citizen scientists live, or to which they have access. Second, it is generally not possible to record all the species occurring in a grid cell; rather, the observed biodiversity is a subset of the actual biodiversity. Both issues (spatially-biased sampling effort and imperfect detection) are hereafter collectively referred to as imperfect sampling.

We used the Southern African Bird Atlas Project data from between June 1, 2007 and Oct. 5, 2013 (second SABAP phase which was still ongoing after our cut-off date). As part of the project, South Africa was divided into 17,339 five-minute grid cells, which for this study were aggregated into 2,025 quarter-degree grid cells (0.25°×0.25°; each cell ca. 625 km^2^). Birders were asked to submit lists of all species that they saw or heard during two hour or longer visits to the five-minute grid cells. By doing so, they also provided information about the species they did not see or hear. In addition, birders were allowed to add further sightings that they made in a grid cell up to five days after the initial list was compiled.

To accommodate imperfect sampling, we used occupancy models. Occupancy models are state-space models that use detection / non-detection data, replicated in space and/or time, to separate species’ occupancy probability from detection probability [35,39]). Furthermore we used a removal design model structure [35] to separate observations made during the initial visits and observations from the following five-day period. We used a set of eight spatially-explicit covariates to predict occupancy and detection probabilities in space (four climate variables that were transformed using a spline model to accommodate potential climate optima in our study species, two vegetation cover variables, human population density, and density of the road network). Full detail about environmental covariates is provided in S1 File in [31,40]. With this type of model, we obtained for each species and grid cell a prediction of conditional occupancy probability, combining information about how likely it was that the species had been overlooked, given the sampling effort, and how likely the species was to occupy a grid cell with similar properties [35]. Importantly, we do not claim to document the functional relationship between the explanatory environmental covariates and potential climatic niches with this correlative extrapolative approach [41,42], but rather use it as a descriptive tool.

### Quaternary refugia in South African biomes

Although information about climates and vegetation composition during the Quaternary in South Africa is fragmented, we found consensual support in the literature for five elements. First, through a combination of lowered CO_2_ atmospheric concentration and increased aridity in the summer rainfall zone, woodland and forest were extirpated from most of South Africa during the Last Glacial Maximum (LGM, ca. 21–18 kyr) [14,40,43–45]. Afromontane forest however persisted in the study region throughout the Quaternary in the form of relictual patches, in particular in the winter and year-round rainfall zones which were locally wetter during the LGM [46,47], and along the Great Escarpment [48]. By contrast, eastern coastal forest was likely completely absent from the study region during the LGM as it retreated northwards [27,44,48]. Forests peaked in extent during the Holocene Altithermal (ca. 10–5 kyr) and are currently in a phase of recession [44]. Second, woodland/savanna refugia are even less accurately delineated, but through analogy with eastern forests and using data from the woodland- and savanna-dwelling Cape buffalo *Syncerus caffer*, we can assume that refugia were located to the North-East, outside of the study region [49]. The Savanna Biome has since been expanding south- and westwards [50]. Elevational gradients and in particular the Great Escarpment have to our knowledge never been mentioned as barriers with a structuring effect on animal biodiversity in the Savanna Biome. Third, the Kalahari in the centre-north of the region experienced several periods of dune activation which are quite poorly understood but indicate repeated periods of unsuitability for savanna bird species throughout the Quaternary and especially after the LGM [51,52]. Thus, the traditional paradigm of a westwards expansion of savanna after the LGM [43] might actually be explained by the influence of the last Heinrich event (ca. 17 kyr) rather than by orbital fluctuations [40,46,51]. During Heinrich events, increased precipitation and decreased temperatures [40] likely helped savanna replace semi-desert vegetation. With savanna encroaching to the east and north during Heinrich events, and fynbos encroaching to west during the LGM [46], the southern regions of the Karoo Biomes therefore appear as the most stable throughout the Quaternary from a semi-desert species perspective [40]. The southern Karoo should thus play the role of an arid refugium. Fourth, the climate experienced by the contemporary Fynbos Biome (defined here as the winter and year-round rainfall area with Mediterranean climate) remained relatively stable throughout the Quaternary, especially in its south-western section [40,46,53,54]. This biome’s particular climate and vegetation exerted a filter against bird colonizers throughout the Quaternary [55], meaning that the Fynbos passerine assemblages are largely isolated from the rest of the region. Fourth, the emergence of the Grassland Biome, another biome with strong filter effect [56], pre-dates Quaternary climate fluctuations by a long time. The Grassland Biome stems from the uplift of the Drakensberg massif (Miocene/Pliocene) creating a rain shadow, and the concomitant development of C4 grasses promoting a regime with frequent fires [57,58]. Out of these five elements we derive the following simple predictions regarding potential Quaternary legacies in passerine distributions:

1. Woodland and Savanna refugia were located to the North-East of the study region. Any species build-up in those biomes should thus translate into northeast to southwest gradients in biodiversity metrics.
2. Arid refugia were located in the south of the Karoo. The Karoo should thus exhibit south to north gradients in biodiversity metrics.
3. The temperate biomes (Fynbos and Grassland) have been relatively stable throughout the Quaternary and their passerine assemblages are largely isolated from the rest of the region by habitat filtering.
4. Forests are now a relictual habitat in the region. The wet climate of the Cape region and the Great Escarpment were important contributors to the maintenance of forest patches in the region. There is nevertheless no quarter degree grid cell exclusively covered by forest. At our scale, forest biodiversity should thus add to the diversity of co-occurring biomes in the grid cells, creating grid cells with relatively high diversity where forest patches occur. In other words, the Forest Biome cannot in our view be adequately studied at the spatial resolution of our study.

### Species richness and species debt, corrected for imperfect sampling

To estimate species richness, we used a parametric bootstrap procedure with 1000 replicates, sampling within the approximate multivariate normal distribution of the parameter estimates of species-specific occupancy models. During each replicate of the bootstrap, we generated species’ presence data based on species- and grid cell-specific occupancy probabilities, and summed over species to obtain species richness.

To estimate species debt, defined as the residual of the regression between species richness and available energy, we used the long term average of the normalized difference vegetation index (NDVI; [59]) as a measure of available energy. NDVI data were obtained from Africa Monitoring of the Environment for Sustainable Development (http://www.amesdsadc.org/latest-products?prod=LtavgNDVI_) on Nov. 21, 2013. Importantly, two major drivers of species richness variation were taken into account before using the residuals of the regression as measure of species debt: habitat heterogeneity and hydric challenge.

Regarding the effect of habitat heterogeneity on species richness, for the same energy availability, more habitat-heterogeneous cells were expected to be artificially more biologically diverse [20,54]. For this reason, we computed species debt separately for each biome. Then, for each grid cell we weighted the estimated biome-specific species debt by the percentage area covered by each biome in the grid cell. If we had not so, grid cells containing a border between two biomes would have artificially appeared more species-rich than expected. Biome coverage data were obtained from Mucina and Rutherford [31] and a grid cell was considered as belonging to a given biome if it was covered at ≥75% by this biome (>0.5% for the marginal Forest and Desert Biomes). Following de Klerk *et al*. [60], we pooled the two semi-desert biomes (Nama Karoo and Succulent Karoo) because despite large differences in floristic assemblages they harbour similar avifaunae. To further correct for habitat heterogeneity in our measure of species debt we also used the elevational range within each grid cell as a proxy for small-scale habitat heterogeneity [20,54]. Elevation data were obtained from Schulze & Horan [61]. For each grid cell we computed the difference between minimum and maximum altitude and we used a fixed effect in the linear models to statistically correct for the additive effect of this measure of topographic heterogeneity on species richness in the grid cells.

Regarding the effect of hydric challenge on grid cell-specific species debt, we predicted that the positive effect of NDVI on bird species richness would be modulated by surface water availability. A discrepancy between NDVI and bird species richness is expected in areas where trees have access to ground water that is not directly available to birds, as is the case in dry savannas. We used the potential to realized evapotranspiration ratio (ETR; [62]) as our measure of hydric challenge. For each grid cell we used a fixed effect in the linear models to statistically correct for the additive effect of ETR on species richness in the grid cells.

The above-described regression was implemented using a mixed regressive spatial autoregressive model (MRSAR). MRSARs [63] estimate the fixed effect of spatially-explicit explanatory covariates while accounting for spatial auto-correlation in the dependent variable using a weight matrix. They provide a measure of spatial auto-correlation (λ parameter) and of the remaining spatial variance (σ parameter). We defined our weight matrix with weight = 1 for grid cells that were neighbours and weight = 0 otherwise. Species debt was then defined as the residuals from the MRSAR (including both the auto-regressive part and the remaining variance). All variables were log-transformed and normalized to a mean of zero and standard deviation of one before analysis in the MRSAR. Statistically non-significant effects were removed before computing species debt.

### Phylogenetic dispersion, corrected for imperfect sampling

An extensive literature of molecular phylogenies and taxonomic classifications (>75 references) was compiled from sources listed in [64,65] and used to inform 15 taxonomic levels. These taxonomic levels were in turn used to compute branch lengths separating species and clades. The resulting taxonomy-based phylogenetic tree is provided in S2 File.

For each simulation in the above-described parametric bootstrap, we applied the usual formula for Net Relatedness Indexes (NRI; [15]), but with a taxonomy-based phylogeny so we hereafter use the phrase “taxonomic dispersion” rather than “phylogenetic dispersion”: ${\mathrm{NRI}}_{i,k}=\frac{{\bar{d}}_{i,k}-{\bar{d}}_{EXP}}{sd(d_{EXP})}$; where ${\bar{d}}_{i,k}$ is the average taxonomic distance among species in grid cell *k* at the *i*th simulation, and ${\bar{d}}_{EXP}$ and *sd*(*d*_*EXP*_) are respectively the average and standard deviation of the expected taxonomic distance for a site with the same species richness. We used the average NRI across 1000 bootstrap replicates as our measure of taxonomic dispersion. Positive NRIs indicated assemblages that were more taxonomically dispersed than expected at random, as predicted if competitive exclusion prevented the coexistence of closely related species [17]. Negative NRIs indicated assemblages that were more taxonomically clumped than expected at random, as predicted if habitat requirements were shared by closely related species [20]. NRI values near zero indicated “taxonomically even” assemblages.

NRIs depend on the definition of the source pool of species available to form the local assemblages (e.g., [66]). By varying that source pool, i.e., by defining different null models of species assemblage, we investigated processes acting at different scales. For the present endeavour, we defined two null models. Null model 1 assumed the source pool to be all the species occurring on the subcontinent. Null model 2 considered the source pool to be the species available locally in a neighbourhood spanning c.100km around the focal grid cell. We hereafter use the notation NRI1 and NRI2 for the net relatedness indexes computed under these two different null models.

Our prediction was that taxonomic dispersion should decrease on a northeast to southwest gradient in the Savanna and Woodland Biomes, reflecting the position of Quaternary refugia in those biomes. We tested this prediction using MRSARs while correcting for two potentially confounding factors. First, NRI is mechanistically linked to species richness, because grid cells with higher richness hold a more complete sample of the source pool and are thus by construction more taxonomically even. Second, NRI is affected by competitive exclusion between related taxa, thus the availability of resources is predicted to influence NRI. In the MRSARs we therefore included the effect of species richness, primary productivity (NDVI; see above), and water availability (ETR; see above), before looking at the spatial variation in NRI by fitting the fixed effects of latitude and longitude (a proxy for distance from mesic refugia). This was performed for all wooded biomes (Savanna, Woodland, Forest, Albany Thicket), for Savanna only, and, as a control, for the Grassland Biome in which we did not expect any effect of distance from mesic refugia. All data analysis and model fitting were performed in R [67].

## Results

### General patterns in species richness and species debt

**Fig 1:**
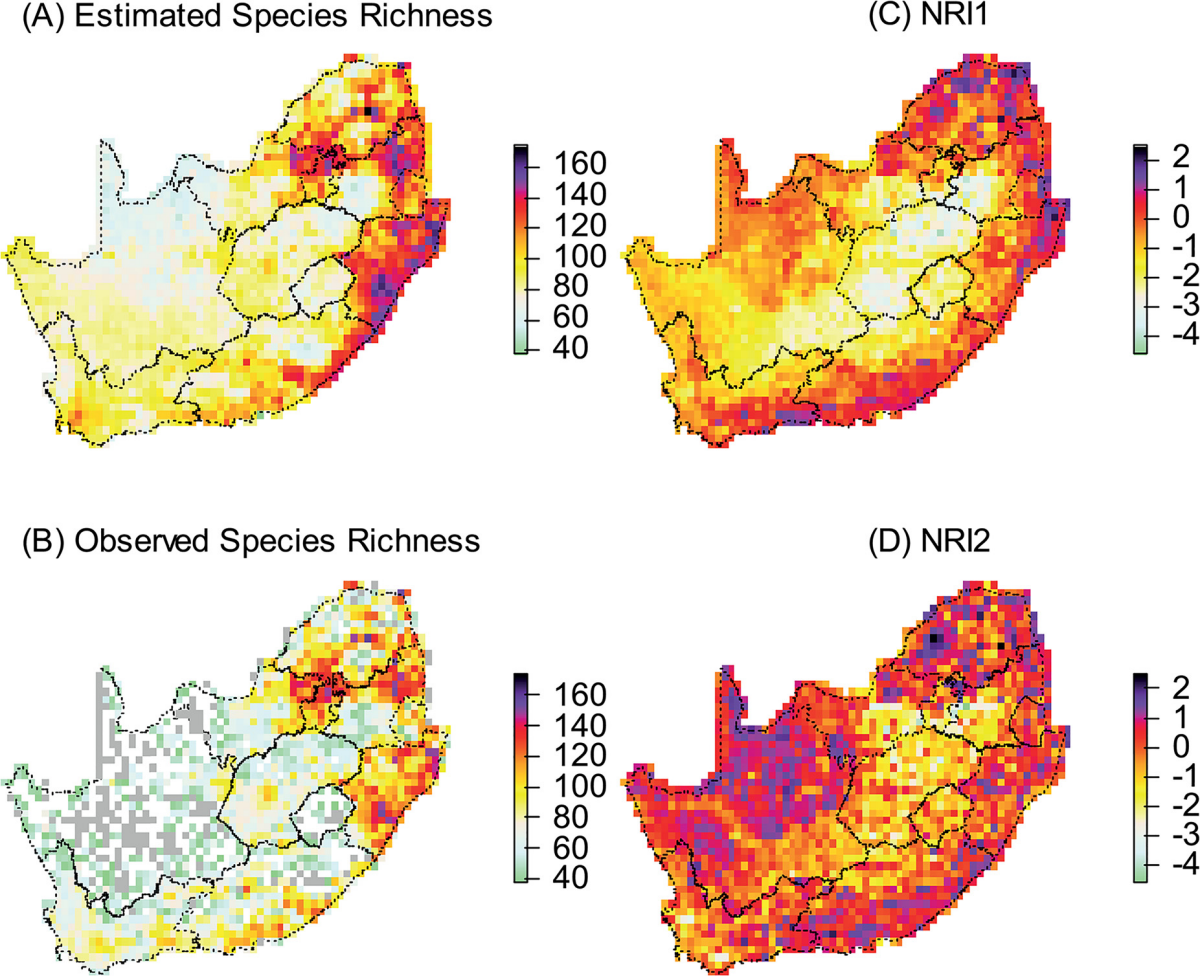
(A): Passerine species richness in South Africa corrected for imperfect sampling. (B): Species richness raw data (number of observed species, not corrected for imperfect sampling). Grey areas were not visited during SABAP2. (C) Taxonomic dispersion under null model 1 (species pool was the whole South African avifauna). (D) Taxonomic dispersion under null model 2 (species pool was the species occurring within a c.100km radius). Values used to plot Fig 1 are in S1 Table.

**Fig 2:**
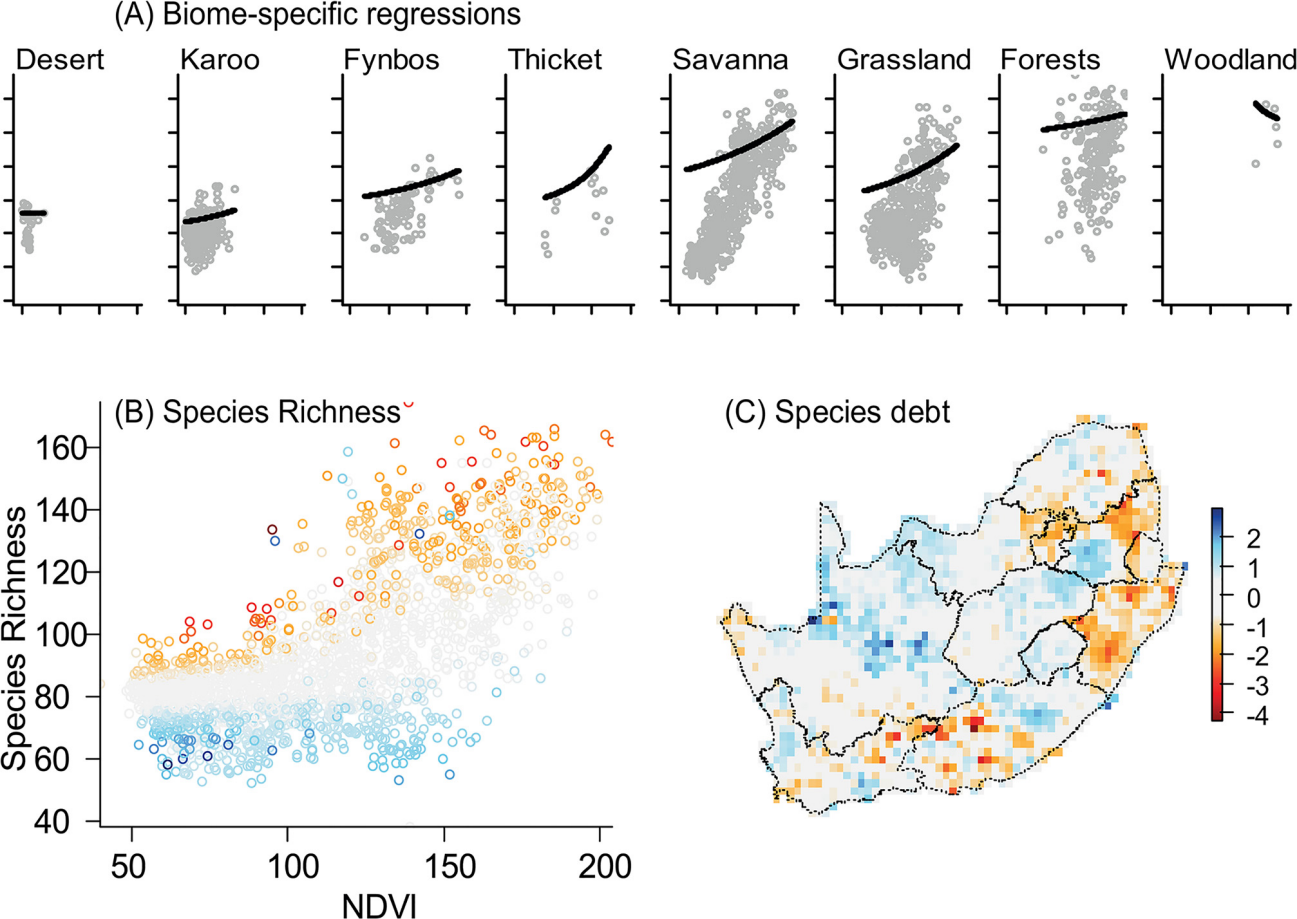
Relationship between passerine species richness and primary productivity (NDVI) in South Africa. (A) Separately in each biome from the westernmost biome to the easternmost one. Grey symbols represent cell-specific estimates and black lines represent the linear regressions after correcting for spatial auto-correlation and the effect of topographic heterogeneity and water availability. Axes labels as in Fig 2B. (B) Across the study region. The colour scheme refers to the species debt legend. (C) Species debt (see *Species debt* in the Material and Methods section for definition and computation details). Negative values (dark blue tones) indicate cells with less species than expected (species debt); positive values (orange tones) indicate cells with more species than expected (species build-up). Values used to plot Fig 2 are in S1 Table.

First, the need to account for imperfect sampling when analysing citizen science data was evidenced by the occurrence of grid cells with low observed species richness but amongst the highest estimated richness (e.g., some of the Swaziland cells; Fig 1A vs. 1B), and conversely grid cells with high observed but average estimated richness (e.g., parts of the Gauteng province where numerous rare species have been recorded due to the high observation pressure; Fig 1A vs. 1B). Species richness was positively correlated to primary productivity, to water availability and, in the Fynbos and Forest Biomes only, to elevational range (Table 1; Fig 2). Between-biome differences suggested a west-east increase in the intercept of the regression of species richness against NDVI and a gentler slope in the Karoo, Grassland and Fynbos Biomes compared to the Savanna Biome (Table 1; Fig 2). Most of the grid cells with more species than expected (Fig 2) contained specific types of forest (Western Cape Afromontane-, Drakensberg Escarpment-, Amatole, Mpumalanga and Eastern Mistbelt-, and KwaZulu-Natal Sand- forests).

**Table 1:** (A) Biome-specific spatially auto-correlated regressions (MRSAR) of passerine species richness against elevational range in the grid cells (Topo HT; left blank if not selected in the final model), water availability (ETR increases with water availability; left blank if not selected in the final model), and primary productivity (NDVI increases with primary productivity). σ and λ respectively estimate the remaining variance and the autocorrelation coefficient. All variables were standardized to a mean of 0 and standard deviation of 1 before analysis. (B) Average and standard deviation of biodiversity metrics (estimated and observed species richness, taxonomic dispersion under the two null models) for each biome.

	(A) MRSAR for species richness						(B) Biome-average and SD of biodiversity metrics			
Biome	Intcp.	Topo HT	ETR	NDVI	σ	λ	SR	SR obs	NRI1	NRI2
Desert*	-0.016 (0.006)		-0.382 (0.079)	0.013 (0.055)	0.532 (0.047)	0.112 (0.019)	83.9 (8.3)	35.0 (12.2)	-1.04 (0.27)	0.59 (0.52)
Karoo	-0.002 (0.004)			0.142 (0.014)	0.796 (0.016)	0.085 (0.006)	81.1 (7.4)	43.8 (21.2)	-1.29 (0.67)	0.15 (0.79)
Fynbos	0.038 (0.012)	0.244 (0.033)		0.204 (0.029)	0.518 (0.014)	0.095 (0.026)	93.3 (12.8)	77.2 (12.6)	-0.21 (0.75)	-0.27 (0.97)
Thicket	0 (0.001)			0.683 (0.057)	0.783 (0.026)	<0.001 (<0.001)	95.6 (16.2)	82.9 (21.1)	0.40 (0.54)	0.54 (0.38)
Savanna	-0.038 (0.009)		-0.128 (0.015)	0.212 (0.129)	0.44 (0.009)	0.107 (0.025)	97.6 (28.1)	77.1 (36.1)	-0.06 (0.82)	0.56 (0.73)
Grassland	0.08 (0.003)		-0.094 (0.007)	0.209 (0.01)	0.529 (0.004)	0.145 (0.004)	91.0 (21.4)	71.3 (27.7)	-1.7 (1.01)	-0.75 (1.05)
Forests*	-0.064 (0.005)	-0.032 (0.008)	0.226 (0.013)	0.092 (0.007)	0.669 (0.013)	0.129 (0.01)	126.9 (21.7)	100.7 (30.9)	0.39 (0.8)	0.48 (0.74)
Woodland	-0.516 (0.1)		0.814 (0.153)	-0.368 (0.122)	0.272 (0.078)	0.745 (0.107)	141.8 (14.7)	116.4 (24.4)	0.69 (0.39)	0.36 (0.7)

* For the Forest and Desert Biomes, regressions were computed across all grid cells where the biome occurred. For other biomes, regressions were implemented for the grid cells with >75% coverage by the biomes.

### General patterns in taxonomic dispersion

Net relatedness indexes obtained using null model 1 (NRI1) indicated high levels of taxonomic clumping in the Grassland Biome, and to a lesser extent the Karoo Biomes (Fig 1C; Table 1). These are species-poor biomes with high rates of endemism and a strong habitat filtering effect acting at relatively high taxonomic levels (e.g., the family Alaudidae is largely restricted to these biomes). As expected, taxonomically even assemblages occurred in species-rich assemblages (Fig 1B vs. 1C; Tables 1 & 2). Net relatedness indexes obtained using null model 2 (NRI2) consistently indicated more taxonomic dispersion than NRI1 (Fig 1D, Table 1), as expected since locally available species are more likely to be represented in local assemblages than species from further afield. In particular the Karoo Biomes appeared taxonomically over-dispersed under null model 2 whereas it was taxonomically clumped under model 1, suggesting that more competitive exclusion occurred between related species in the arid Karoo than in other biomes.

**Table 2:** Spatially auto-correlated regressions of taxonomic dispersion (NRI1) against species richness SR, latitude, longitude, water availability (ETR increases with water availability), and primary productivity (NDVI increases with primary productivity). σ and λ respectively estimate the remaining variance and the autocorrelation coefficient. All variables were standardized to a mean of 0 and standard deviation of 1 before analysis.

	intcp	SR	Lat.	Long.	Long*Lat	ETR	NDVI	ETR*NDVI	σ	λ
All Wooded Biomes										
estimates	-0.098	-0.145	0.005	0.238	0.200	-0.344	0.487	0.082	0.604	0.340
SE	0.04	0.043	0.039	0.061	0.032	0.057	0.062	0.033	0.016	3E-05
Savanna only										
estimates	-0.145	-0.216	0.18	0.113	0.002	-0.368	0.544	0.131	0.492	0.071
SE	0.038	0.042	0.037	0.071	0.042	0.057	0.083	0.026	0.016	0.0004
Grassland										
estimates	0.191	0.053	0.003	-0.085	-0.009	0.056	0.111	-0.175	0.622	0.142
SE	0.043	0.04	0.041	0.082	0.034	0.065	0.071	0.032	0.021	0.0001

### Northeast to southwest gradients in the wooded biomes

Across the grid cells that did not overlay with the Forest or Karoo Biomes, species debt increased on a north-east to south-west gradient (MRSAR slope estimates: longitude: -0.028 ± SE 0.016; latitude: 0.16 ± SE 0.16; interaction: 0.002 ± SE 0.012). Taxonomic dispersion in the wooded biomes also increased on a north-east to south-west gradient, but only after statistically correcting for the effects of species richness, primary productivity, and water availability (Table 2). In other words, contemporary water and energy availability could not explain the high level of taxonomic dispersion observed in the north-easternmost corner of the study region (Fig 1C).

### Southern Karoo refugium?

The far south of the Nama Karoo Biome had more species than expected, suggesting species-build-up (Fig 2). But in addition, the Succulent Karoo was on average less species-rich than the Nama Karoo, leading to species debt increasing from east to west when pooling these two biomes, rather than south to north as we predicted (Fig 2). There was however no statistically significant latitudinal or longitudinal gradient in taxonomic dispersion in the Karoo. We thus found limited support for the occurrence of Quaternary legacies in the Karoo passerine assemblages.

## Discussion

### Macroecological and biogeographic inference

Using crowdsourced data and occupancy models, we found a northeast to southwest gradient in both species debt (the residuals of the regression between species richness and available energy) and taxonomic dispersion (NRI1) of the passerine assemblages of South African wooded biomes, especially the assemblages of the Savanna Biome. We interpret these gradients as a consequence of the location of Quaternary mesic refugia. This interpretation is consistent with results from previous analyses using only the relictual patches of Afromontane and coastal forests [44,47,48], which we now extend to the whole ensemble of wooded biomes. The Kalahari section of the Savanna Biome, in the centre-north of the study region, appears particularly interesting in that respect. We found that this region of arid savanna was poorer in passerine species than expected based on contemporary average environmental conditions (Fig 2). Yet, throughout most of the Quaternary, the Kalahari was wetter than today [40,46]. Thus we would expect that the region inherited species from its mesic past, but we observe the exact opposite. This apparent contradiction is resolved when the abundant evidence for periodic mobilisation of sand dunes in the Kalahari [51,52] is taken into consideration. During these periods that are associated with Heinrich events (change in atmospheric circulation linked to the sudden release into the Atlantic ocean of “iceberg armadas” from receding icesheets [68]), the Kalahari was most probably completely inhospitable for savanna bird species. In brief, the Kalahari experienced wide fluctuations in its environment since the LGM, which correlates with its reduced avian diversity today. A congruent piece of evidence for Quaternary legacies in contemporary passerine distributions was the taxonomic dispersion of local assemblages in the north-easternmost corner of the study area (closest to purported woodland refugia) as well as in the Western Cape’s Knysna area (a regionally important Afromontane refugium). When distance from the north-eastern refugia increased, assemblages were increasingly clustered, i.e., more and more clades were lacking. Combined with the species debt results, this pattern suggests a filter acting against less mobile species and clades.

Yet, the distances involved in our study region are moderate (order of magnitude 1000km) compared to what can be assumed of the dispersal abilities of South African passerines. For this reason, our results suggest philopatric syndromes rather than physiological constraint as the proximal cause of Quaternary legacies. This is to our knowledge the first time that this hypothesis is suggested.

Our interpretation of Quaternary legacies as the long-term effect of philopatric syndromes implies that the intensity of those syndromes is clade-specific. Yet mobility and dispersal are typically considered evolutionary labile in birds, by analogy with the evolutionary lability of seasonal migration [69], i.e., the fact that long-distance migration behaviour has been lost and found numerous times during avian evolution [70,71]. However we argue that if we distinguished migration behaviour (whether individuals change territory after breeding) and migration distance (whether the seasonal territories are widely separated), then we would find a strong signal of phylogenetic conservatism in migration behaviour. As highlighted in the introduction, in the African-Palearctic flyway, whole bird families have proven unable to evolve long-distance migration and are, possibly consequently, absent or sparsely represented in the Western Palearctic [72]. On the other hand migratory restlessness (Zugunruhe, a behaviour associated with migration) is expressed even by resident populations of migratory clades [73]. Lastly, dispersal and migration are quite different phenomena, and phylogenetic conservation of dispersal tendencies seems less controversial [21,22], be it only because adaption to ecological niches entails morphological and behavioural modifications with consequences on mobility. In short, several lines of evidence corroborate our interpretation of the northeast-southwest gradient in taxonomic dispersion of passerine assemblages could be a Quaternary legacy mediated by philopatric syndromes.

Lastly, the east-west gradient in species richness and taxonomic dispersion in the Karoo Biomes was not expected by us. It may suggest an eastern, dry savanna origin for some of the Karoo species, with the transition from summer rainfall in the Nama Karoo to winter rainfall in the Succulent Karoo acting as a filter. Species with hard-wired breeding phenologies that are adapted to summer rainfall probably have lower population viability in the Succulent Karoo (e.g., the widespread rufous-eared warbler *Malcorus pectoralis*, chat flycatcher *Bradornis infuscatus*, and Southern anteating-chat *Myrmecocichla formicivora* all breed during the austral spring and show a marked decrease in abundance in the winter rainfall zone; [36]). The above elements suggest that including the seasonality and predictability of rainfall would improve species distribution predictions in the Karoo Biomes.

### Method discussion

Occupancy models account for imperfect detection and spatially-biased sampling effort, allowing to take advantage of the abundant, fine-resolution, but fragmented information provided by citizen science projects. However, the major drawback of the approach implemented in this study, and others, is the reliance on environmental covariates. When the species distribution data are sparse, this can lead to inaccurate extrapolations, for two main reasons: 1) For hard-to-detect species, occupancy models require relatively dense non-detection data to indicate species absence with high confidence; combined with the usual biases associated with extrapolating from the centre of an environmental gradient to its extremes, this can potentially lead to poor predictions in grid cells with extreme covariate values but few or no atlas data (see also [74,75]). 2) The spatially-explicit environmental covariates, even if we included more of them than previous modelling efforts, may not effectively capture the variation in biodiversity if the latter is unrelated to contemporary conditions. In other words, grid cells affected by Quaternary legacies but poorly sampled by atlas contributors would not be identified as such, reducing the power to detect Quaternary legacies. We partly addressed those issues by using the conditional rather than unconditional formulation of occupancy probabilities (see [35] pp 97–99 for technical details). The drawback of this approach is that each observed species, even if observed just once, is considered as having a 100% occupancy probability, which may not deal well with the issue of vagrancy in birds. Incorporating auto-correlation structures such as kriging would lessen the reliance on environmental covariates in the models, but these methods are even more sensitive to data sparseness than our approach and generally tend to “over-smooth” parameter estimates where data is sparse [76]. A last caveat is that the species debt pattern that we report was possibly inflated by the protection afforded to the species-rich eastern savannas and woodlands by the Kruger conservation area. This confounding factor is however not as worrisome in passerines as it is in larger-bodied clades, because the former can generally subsist in fragmented habitat, and numerous smaller reserves occur throughout the Savanna Biome. With the above caveats in mind, our macroecological and biogeographic inferences still hold in our view because the observed gradients span across some of the most data rich areas in the region.

### Conservation perspectives

Quaternary mesic and humid refugia have been identified as regions of high conservation value, because they hold more species per unit of habitat and have proven able to preserve them during past climate fluctuations [11,13]. Even though past climate fluctuations are not good surrogates for future ones [77], Quaternary mesic refugia will play an important role as a source of diversity for future redistributions. It is thus critical to identify them, in order to enhance their connectivity with areas forecast to become new centres of diversity under future conditions. In this study we make the case for combining two biodiversity metrics, species debt and taxonomic dispersion, in order to identify the location of Quaternary refugia within ecologically homogeneous biomes. In species-rich, mesic biomes, refugia should have more species than expected based on contemporary environmental conditions and more dispersed assemblages than the average; in species-poor, arid biomes, refugia should have less species than expected and more clumped assemblages than the average (see however our result pertaining to the possible southern Karoo refugium). Our analysis further pinpoints the risk of flawed inference linked to the occurrence of relictual small patches of species-rich habitat within grid cells, such as forest in our study region, which because of their small extent do not contribute much to the average NDVI of the grid cells, but harbour a contrasting and rich suite of specialist species. Our results also confirm that more than one metric should be used to prioritize conservation objectives at the sub-continental or continental scale [78,79]. In particular the high level of NRI1 taxonomic clumping in species-poor areas was associated with the presence of unique clades which would be counter-selected if conservation decisions were based on species richness alone [80]. A more quantitative study is also warranted for decision support tools that balance the irreplaceability of mesic refugia and the more widespread consequences that diversity loss in more common, representative landscapes would incur [56]. Lastly, NRI2 could be used as a metric for beta diversity. In order to encompass a representative sample of the landscape diversity, protected areas in regions with low NRI2 (such as grasslands) would not need to be as large as those in regions with high NRI2 (such as savannas).

In conclusion, our study, which is to our knowledge the first to correct species distribution models for imperfect sampling in a whole order of vertebrates and at the sub-continental scale, found significant structure in the residuals of the regression of species richness against energy and water availability, which was congruent with similar gradients that we report for taxonomic dispersion of local assemblages. We suggest the intriguing interpretation of these patterns as Quaternary legacies mediated by philopatric syndromes (behavioural rather than physical constraints on dispersal).

## Supporting Information

S1 File Taxonomic tree. (PDF)

S2 File Environmental covariates. (PDF)

S1 Table Estimate values used to plot main text Figs 1 & 2. (XLSX)
