# Supplementary material for: Departures from the Energy-Biodiversity Relationship in South African Passerines: Are the Legacies of Past Climates Mediated by Behavioral Constraints on Dispersal?
Source: PLoS One. 2015 Jul 24;10(7):e0133992. doi: 10.1371/journal.pone.0133992 (PMC4514734; doi:10.1371/journal.pone.0133992)
Supplement: S1 File — (PDF) [file pone.0133992.s001.pdf]

Departures from the energy-biodiversity relationship in South African passerines: are the legacies of past climates mediated by behavioral constraints on dispersal? by Guillaume Péron and Res Altwegg

S1 File

Fig. S1.1: Taxonomic tree. Two representations of the same tree. Only some of the species' names could be displayed due to space limitations.

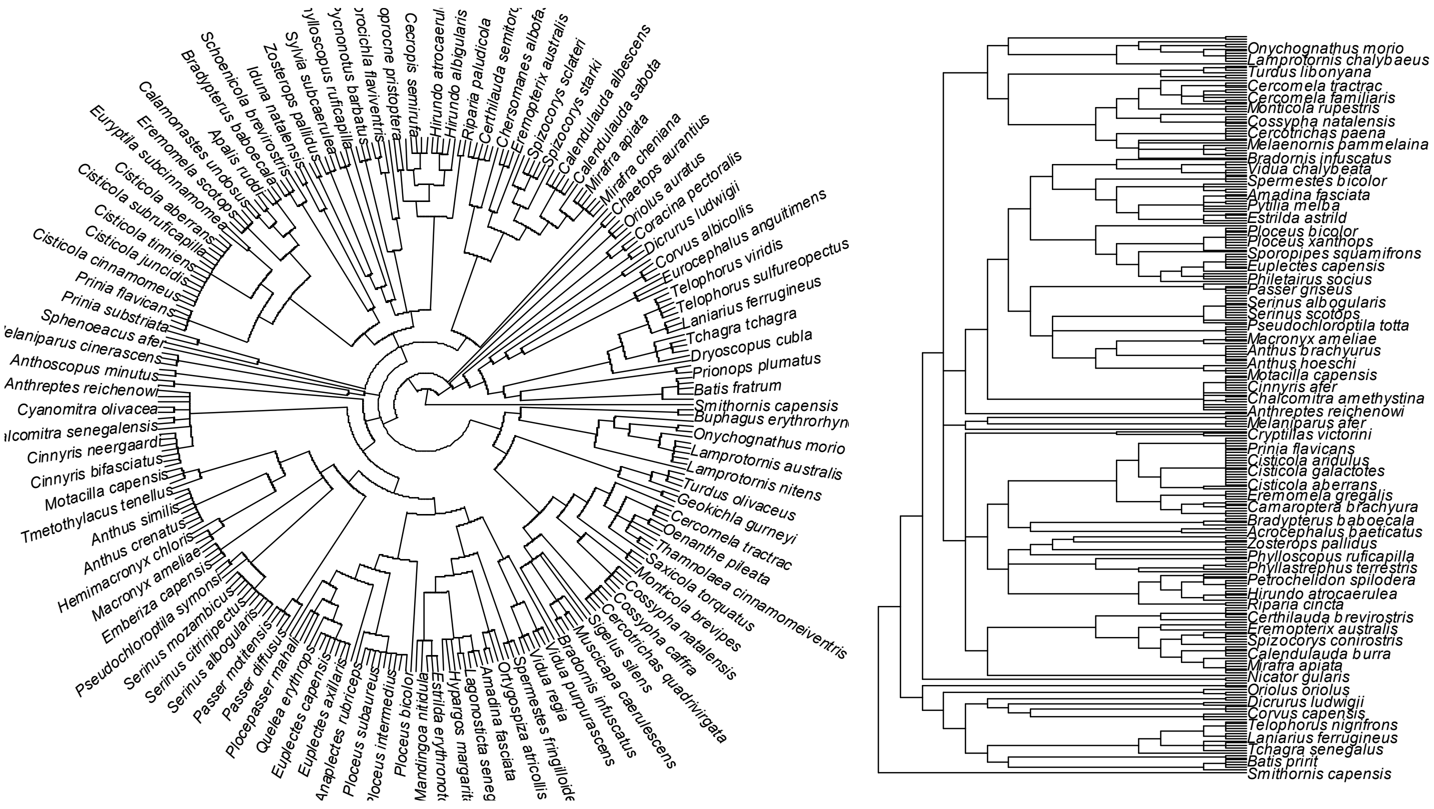

Text S1.2: Taxonomic tree in Newick format

(Smithornis_capensis:17,(((((((Platysteira_peltata:2,(Batis_capensis:1,Batis_fratrum:1,Batis_molitor:1,Batis_pririt:1):1):9,(Prionops_plumatus:1,Prionops_retzii:1):10):1,(Nilaus_afer:5,((Dryoscopus_cubla:2,(Tchagra_senegalus:1,Tchagra_australis:1,Tchagra_tchagra:1):1,Malaconotus_blanchoti:2):2,((Laniarius_aethiopicus:1,Laniarius_ferrugineus:1,Laniarius_atrococcineus:1):1,(Telophorus_zeylonus:1,Telophorus_sulfureopectus:1,Telophorus_olivaceus:1,Telophorus_nigrifrons:1,Telophorus_viridis:1):1):2):1):7):1,((((Lanius_collaris:2,Corvinella_melanoleuca:2,Eurocephalus_anguitimens:2):8,(Corvus_capensis:1,Corvus_albus:1,Corvus_albicollis:1):9):1,(Trochocercus_cyanomelas:2,Terpsiphone_viridis:2):9):1,(Dicrurus_ludwigii:1,Dicrurus_adsimilis:1):11):1):1,((Coracina_caesia:1,Coracina_pectoralis:1):1,Campephaga_flava:2):12):1,(Oriolus_oriolus:1,Oriolus_auratus:1,Oriolus_larvatus:1):14):1,((Chaetops_frenatus:1,Chaetops_aurantius:1):14,(((Nicator_gularis:12,((((((Mirafra_passerina:1,Mirafra_cheniana:1,Mirafra_africana:1,Mirafra_rufocinnamomea:1,Mirafra_apiata:1,Mirafra_fasciolata:1):1,Heteromirafra_ruddi:2):1,(Calendulauda_sabota:1,Calendulauda_africanoides:1,Calendulauda_burra:1,Calendulauda_albescens:1,Calendulauda_barlowi:1):2):1,(Calandrella_cinerea:3,((Spizocorys_starki:1,Spizocorys_conirostris:1,Spizocorys_fringillaris:1,Spizocorys_sclateri:1):1,Galerida_magnirostris:2):1):1):1,(Pinarocorys_nigricans:4,(Eremopterix_australis:1,Eremopterix_leucotis:1,Eremopterix_verticalis:1):3):1):2,(Chersomanes_albofasciata:4,(Certhilauda_curvirostris:1,Certhilauda_brevirostris:1,Certhilauda_semitorquata:1,Certhilauda_subcoronata:1,Certhilauda_chuana:1):3):3):5):1,((((Riparia_paludicola:1,Riparia_cincta:1):4,(((Ptyonoprogne_fuligula:2,(Hirundo_albigularis:1,Hirundo_smithii:1,Hirundo_dimidiata:1,Hirundo_atrocaerulea:1):1):1,((Cecropis_cucullata:1,Cecropis_abyssinica:1,Cecropis_semirufa:1,Cecropis_senegalensis:1):1,Petrochelidon_spilodera:2):1):1,(Psalidoprocne_pristoptera:2,Pseudhirundo_griseopyga:2):2):1):6,((Andropadus_importunus:5,(Chlorocichla_flaviventris:2,(Phyllastrephus_terrestris:1,Phyllastrephus_flavostriatus:1):1):3):2,(Pycnonotus_barbatus:1,Pycnonotus_nigricans:1,Pycnonotus_capensis:1):6):4,(Phylloscopus_ruficapilla:10,((Sylvia_nigricapilla:1,Sylvia_layardi:1,Sylvia_subcaerulea:1):8,((Zosterops_senegalensis:1,Zosterops_pallidus:1,Zosterops_pallidus:1):7,(Turdoides_bicolor:1,Turdoides_jardineii:1):7):1):1):1):1,(((Iduna_natalensis:3,(Acrocephalus_baeticatus:1,Acrocephalus_gracilirostris:1):2):7,(Schoenicola_brevirostris:2,(Bradypterus_barratti:1,Bradypterus_sylvaticus:1,Bradypterus_baboecala:1):1):8):1,((((Apalis_thoracica:1,Apalis_flavida:1,Apalis_ruddi:1):2,((Camaroptera_brachyura:1,Camaroptera_brachyura:1):1,(Calamonastes_undosus:1,Calamonastes_fasciolatus:1):1):1):1,(Eremomela_icteropygialis:1,Eremomela_scotops:1,Eremomela_gregalis:1,Eremomela_usticollis:1):3):2,(((Euryptila_subcinnamomea:2,Malcorus_pectoralis:2):2,(Cisticola_erythrops:1,Cisticola_aberrans:1,Cisticola_chiniana:1,Cisticola_rufilatus:1,Cisticola_subruficapilla:1,Cisticola_lais:1,Cisticola_galactotes:1,Cisticola_tinniens:1,Cisticola_natalensis:1,Cisticola_fulvicapilla:1,Cisticola_juncidis:1,Cisticola_aridulus:1,Cisticola_textrix:1,Cisticola_cinnamomeus:1,Cisticola_ayresii:1):3):1,(Prinia_subflava:1,Prinia_flavicans:1,Prinia_maculosa:1,Prinia_hypoxantha:1,Prinia_substriata:1):4):1):5):1):1,((Sylvietta_rufescens:2,Cryptillas_victorini:2):4,Sphenoeacus_afer:6):7):1,(Stenostira_scita:13,((Melaniparus_niger:1,Melaniparus_cinerascens:1,Melaniparus_afer:1):11,(Anthoscopus_caroli:1,Anthoscopus_minutus:1):11):1):1,((Promerops_gurneyi:1,Promerops_cafer:1):12,((Anthreptes_reichenowi:2,Hedydipna_collaris:2,Anthobaphes_violacea:2,(Cyanomitra_olivacea:1,Cyanomitra_veroxii:1):1,(Chalcomitra_amethystina:1,Chalcomitra_senegalensis:1):1,Nectarinia_famosa:2,(Cinnyris_chalybeus:1,Cinnyris_neergaardi:1,Cinnyris_afer:1,Cinnyris_mariquensis:1,Cinnyris_bifasciatus:1,Cinnyris_talatala:1,Cinnyris_fuscus:1):1):10,((((((Motacilla_capensis:1,Motacilla_clara:1,Motacilla_aguimp:1):1,Tmetothylacus_tenellus:2):5,((Anthus_cinnamomeus:1,Anthus_hoeschi:1,Anthus_similis:1,Anthus_leucophrys

:1,Anthus_lineiventris:1,Anthus_crenatus:1,Anthus_brachyurus:1,Anthus_caffer:1):5,(Hemimacronyx_chloris:2,(Macronyx_capensis:
1,Macronyx_croceus:1,Macronyx_ameliae:1):1):4):1):2,(Emberiza_impetuani:1,Emberiza_tahapisi:1,Emberiza_capensis:1,Emberiza_
flaviventris:1):8,((Pseudochloroptila_totta:1,Pseudochloroptila_symonsi:1):1,Alario_alario:2,(Serinus_canicollis:1,Serinus_mozambic
us:1,Serinus_scotops:1,Serinus_atrogularis:1,Serinus_citrinipectus:1,Serinus_sulphuratus:1,Serinus_flaviventris:1,Serinus_albogularis:
1,Serinus_leucopterus:1,Serinus_gularis:1):1):7):1,((Passer_motitensis:1,Passer_melanurus:1,Passer_griseus:1,Passer_diffusus:1):1,Pet
ronia_superciliaris:2):8):1,(((((Bubalornis_niger:4,((Plocepasser_mahali:2,Philetairus_socius:2,Quelea_quelea:2):1,(Quelea_erythrops:
2,(Euplectes_orix:1,Euplectes_afer:1,Euplectes_capensis:1,Euplectes_albonotatus:1,Euplectes_ardens:1,Euplectes_axillaris:1,Euplecte
s_progne:1):1):1):1):1,Sporopipes_squamifrons:5):1,(Anaplectes_rubriceps:2,(Ploceus_ocularis:1,Ploceus_capensis:1,Ploceus_subaure
us:1,Ploceus_xanthops:1,Ploceus_xanthopterus:1,Ploceus_intermedius:1,Ploceus_velatus:1,Ploceus_cucullatus:1,Ploceus_bicolor:1):1)
:4):1,Amblyospiza_albifrons:7):3,(((((Coccopygia_melanotis:2,Mandingoa_nitidula:2,(Estrilda_perreini:1,Estrilda_astrild:1,Estrilda_e
rythronotos:1):1):3,(Uraeginthus_angolensis:2,Granatina_granatina:2,Hypargos_margaritatus:2,(Pytilia_melba:1,Pytilia_afra:1):1,(Lag
onosticta_senegala:1,Lagonosticta_rubricata:1,Lagonosticta_rhodopareia:1):1):3):1,((Amadina_fasciata:1,Amadina_erythrocephala:1):
1,Sporaeginthus_subflavus:2,Ortygospiza_atricollis:2):4):1,(Spermestes_cucullatus:1,Spermestes_bicolor:1,Spermestes_fringilloides:1
):6):2,(((Vidua_macroura:1,Vidua_paradisaea:1,Vidua_regia:1):2,(Vidua_chalybeata:1,Vidua_funerea:1,Vidua_purpurascens:1):2):3,
Anomalospiza_imberbis:6):3):1):1):1):1):1,(((((Bradornis_pallidus:5,((Bradornis_infuscatus:1,Bradornis_mariquensis:1):1,(Muscicapa
_adusta:1,Muscicapa_caerulescens:1):1,Myioparus_plumbeus:2):3,Melaenornis_pammelaina:5,Sigelus_silens:5):1,(Cercotrichas_cory
phaeus:1,Cercotrichas_signata:1,Cercotrichas_quadrivirgata:1,Cercotrichas_paena:1,Cercotrichas_leucophrys:1):5):1,(((Cossypha_caff
ra:1,Cossypha_humeralis:1,Cossypha_heuglini:1,Cossypha_natalensis:1,Cossypha_dichroa:1):3,Pogonocichla_stellata:4):2,((Monticol
a_brevipes:1,Monticola_explorator:1,Monticola_rupestris:1):4,((Saxicola_torquatus:3,((Myrmecocichla_formicivora:1,Myrmecocichla
_arnotti:1):1,Thamnolaea_cinnamomeiventris:2,Cercomela_familiaris:2,(Oenanthe_monticola:1,Oenanthe_pileata:1):1):1,(Cercomela_
sinuata:1,Cercomela_schlegelii:1,Cercomela_tractrac:1):2):1,Saxicola_bifasciatus:4):1):1):1):4,((Geokichla_guttata:1,Geokichla_gurn
eyi:1):3,(Psophocichla_litsitsirupa:2,(Turdus_libonyana:1,Turdus_olivaceus:1,Turdus_smithi:1):1):2):7):1,(((Creatophora_cinerea:6,(((
(Lamprotornis_nitens:1,Lamprotornis_chalybaeus:1,Lamprotornis_mevesii:1,Lamprotornis_australis:1,Lamprotornis_corruscus:1):1,S
preo_bicolor:2):2,(Onychognathus_morio:1,Onychognathus_nabouroup:1):3):1,Cinnyricinclus_leucogaster:5):1):5,(Buphagus_erythro
rhynchus:1,Buphagus_africanus:1):10):1):2):1):1):1);
