# Supplementary material for: Departures from the Energy-Biodiversity Relationship in South African Passerines: Are the Legacies of Past Climates Mediated by Behavioral Constraints on Dispersal?
Source: PLoS One. 2015 Jul 24;10(7):e0133992. doi: 10.1371/journal.pone.0133992 (PMC4514734; doi:10.1371/journal.pone.0133992)
Supplement: S2 File — (PDF) [file pone.0133992.s002.pdf]

Departures from the energy-biodiversity relationship in South African passerines: are the legacies of past climates mediated by behavioral constraints on dispersal? by Guillaume Péron and Res Altwegg

S2 File

Spatially-explicit environmental covariates

We used a set of eight spatially-explicit covariates to predict spatial variation in occupancy and detection probabilities.

A) Four climate variables were selected following Huntley et al. (2006): the coldest average monthly temperature, the warmest average monthly temperature, the ratio of potential to realized evapotranspiration (ETR; a measure of vegetation cover and hydric stress), and the intensity of the wet season. The climate data were first standardized to a mean of 0 and a standard error of 1 and then processed using a regression spline [2] with two knots, in order to accommodate the potential existence of climate optima for our study species.

Fig. S2.1: The four climate covariates plotted at the quarter degree resolution. Temperatures (Temp.) are in °C, and the realized to potential evapotranspiration ratio (ETR) and wet season intensity have no unit.

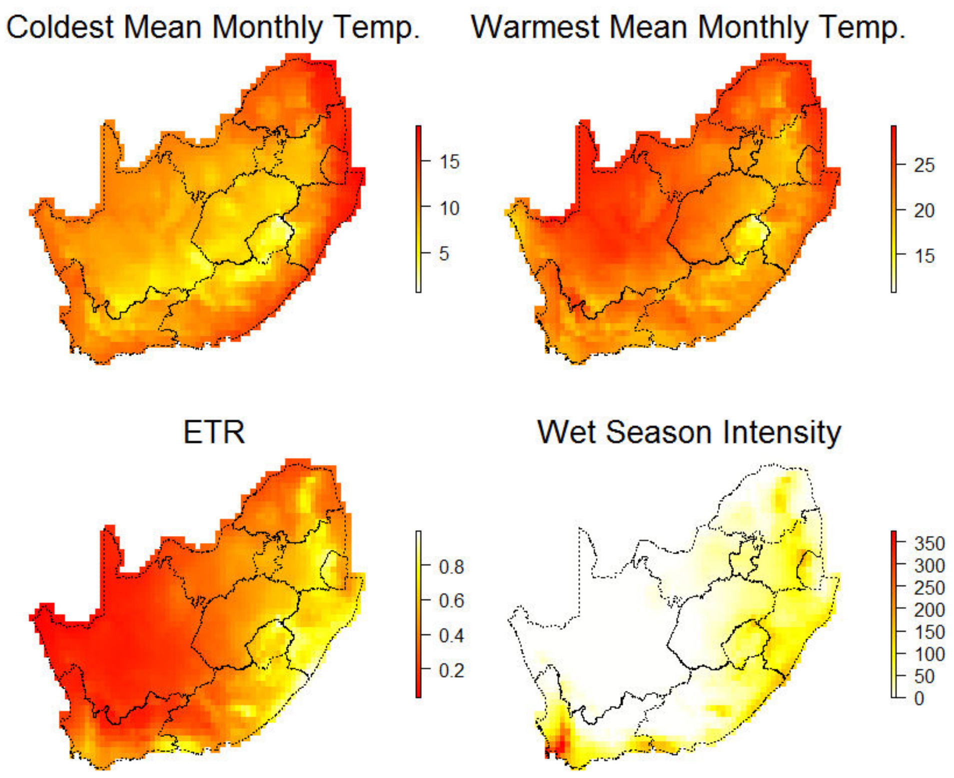

B) Two vegetation cover variables were obtained from Mucina and Rutherford (2011): the first variable was whether more than 1% of the cell had large trees (e.g., evergreen forest, woodland), and the second variable was whether more than 1% of the cell was covered by lower or sparser woody vegetation (e.g., fynbos, bushveld). This was meant to model the need of the study species for specific structural features in their habitat, e.g., absence of trees for steppe-breeding birds which perceive trees as potential vantage points for predators. These two binary variables did not correlate to the climate variables.

Fig. S2.2: The two habitat structure covariates (binary covariates) plotted at the quarter degree resolution.

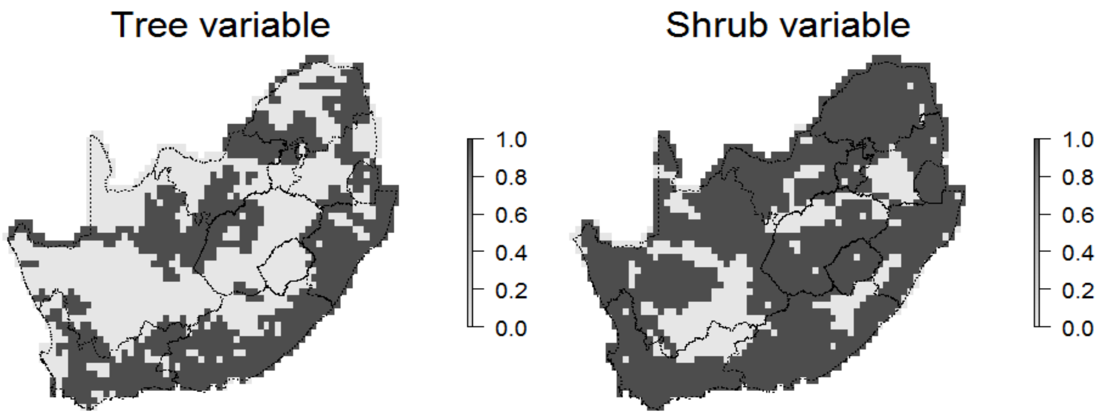

C) The last two variables, human population density and road density, captured both the degree of anthropization of the habitat and the intensity of primary production (since human density centers are located in productive areas). Based on preliminary analyses and on theoretical expectations regarding the shape of the functional response of species to increase in primary productivity and disturbance, these variables were log-transformed after adding 1, and then standardized to a mean of zero and SD of one.

Fig. S2.3: Human population density and density of the road network, log transformed and plotted at the quarter degree resolution. Human population density is in people per km2 and road network density is an index proportional to the length of paved roads in each grid cell.

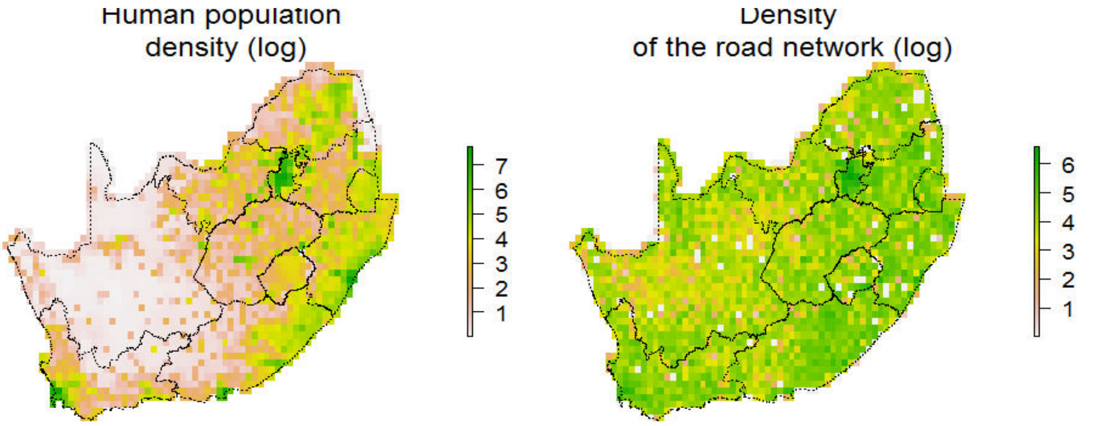

Literature cited in this Appendix

1. Huntley B, Collingham YC, Green RE, Hilton GM, Rahbek C, Willis SG (2006) Potential impacts of climatic change upon geographical distributions of birds. Ibis 148: 8–28.

2. Crainiceanu C, Ruppert D, Wand M (2005) Bayesian Analysis for Penalized Spline Regression Using Win BUGS. J Stat Softw 14: 1–24.

3. Mucina L, Rutherford MC, editors (2011) The vegetation of South Africa, Lesotho and Swaziland (reprint). Pretoria: South African National Biodiversity Institute.
